# Cardiometabolic adaptations in the cave nectar bat *Eonycteris spelaea*

**DOI:** 10.1038/s42003-026-09792-8

**Published:** 2026-03-10

**Authors:** Fan Yu, Akshamal M. Gamage, Myu Mai Ja Kp, Randy Foo, Ying-Hsi Lin, Lijin Wang, Chee Jian Pua, Wharton O. Y. Chan, Gustavo E. Crespo-Avilan, Edgar M. Pena, Lewis Z. Hong, Aditya Iyer, Sujoy Ghosh, Elisa A. Liehn, Jean-Paul Kovalik, Lin-Fa Wang, Chrishan J. Ramachandra, Derek J. Hausenloy

**Affiliations:** 1 National Heart Research Institute Singapore, National Heart Centre Singapore, Singapore, Singapore; 2 Cardiovascular & Metabolic Disorders Programme, Duke-NUS Medical School, Singapore, Singapore; 3 Yong Loo Lin School of Medicine, National University of Singapore, Singapore, Singapore; 4 Programme in Emerging Infectious Diseases, Duke-NUS Medical School, Singapore, Singapore; 5 Centre for Computational Biology, Duke-NUS Medical School, Singapore, Singapore; 6 SingHealth Experimental Medicine Centre and National Large Animal Research Facility, Singapore, Singapore; 7 Paratus Sciences Singapore Pte Ltd, Singapore, Singapore; 8 Excelra, NSL Arena, Uppal, Hyderabad, India; 9 Pennington Biomedical Research Center, Baton Rouge, LA USA; 10 The Hatter Cardiovascular Institute, University College London, London, UK

**Keywords:** Molecular evolution, Non-model organisms

## Abstract

Bats are the only mammals capable of powered flight, a physiologically demanding process requiring substantial cardiac energy expenditure. Here, we investigate how hearts of the cave nectar bat *Eonycteris spelaea* are adapted to meet these extreme demands. Transcriptomic profiling reveals enriched signatures of oxidative phosphorylation and fatty acid metabolism in bat hearts, distinct from mouse and human counterparts. Metabolomics analyses corroborate these findings, identifying distinct acylcarnitine profiles and elevated tricarboxylic acid cycle intermediates. Anatomically, bats have relatively larger hearts with increased mitochondrial and vascular densities along with prominent perivascular adipocytes. Echocardiography reveals superior cardiac reserve in bats, with enhanced contractile responses under dobutamine stress. Notably, isolated bat cardiomyocytes resist angiotensin II-induced hypertrophy and mitochondrial dysfunction. These integrated adaptations likely support high-energy flight while preserving cardiac function under stress. Insights from bat cardiac physiology may provide valuable information on cardioprotective mechanisms with potential application across species.

## Abstract (web summary)

Multimodal analyses of cave nectar bat hearts reveal transcriptional, metabolic, and structural adaptations underlying a highly functional, stress-resistant heart capable of sustaining the extreme demands of powered flight.

## Introduction

Bats are a unique group of mammals with notable characteristics such as extended lifespan, resistance to viral infections and cancer, and powered flight^1,2^. Compared to terrestrial mammals, bats experience significantly higher energy expenditures, particularly during active flight. For instance, certain frugivorous bat species can increase their basal metabolic rates by up to 15-fold and achieve heart rates as high as 900 beats per minute^3^. To accommodate these high energy requirements, bats have undergone natural selection favouring mitochondrial and nuclear genes involved in oxidative phosphorylation^4^.

The cardiovascular system of bats has evolved several distinctive features to facilitate their unique lifestyle. Bats have the largest relative heart size among mammals, supporting their enhanced aerobic metabolism and the high oxygen demands of flight^5^. Comparative studies show that bat cardiomyocytes contain a higher mitochondrial volume fraction, increased crista density, a wider T-tubule system, and greater lipid body concentrations compared to other small mammals like hamsters and rats^6,7^. These structural adaptations suggest enhanced metabolic efficiency and energy storage capabilities that contribute to their exceptional exercise tolerance. Notably, their characteristic inverted resting position subjects the heart to sustained biomechanical stress, thereby inducing injury^8^.

Despite these insights into the structural characteristics of bat hearts, a comprehensive understanding of how they respond to extreme physiological demands remains critically unexplored. Existing research has primarily focused on gross anatomy and ultrastructure, which leaves a significant gap in knowledge regarding how various bat species maintain cardiac function under sustained metabolic challenges. Given the high energy demands of flight, we hypothesise that bat hearts have evolved specific cardiometabolic adaptations to preserve myocardial energetics during prolonged stress. Our findings reveal evolutionarily conserved enrichment in energy-related pathways in bat hearts, along with enhanced metabolic capacity through distinct metabolite signatures and structural features, and functional studies confirming stress resilience in bat cardiomyocytes. These integrated adaptations likely support high-energy flight while preserving cardiac function under stress in bats.

## Results

### Bat hearts exhibit enriched transcriptomic signatures related to energy production and metabolism

**Fig. 1:**
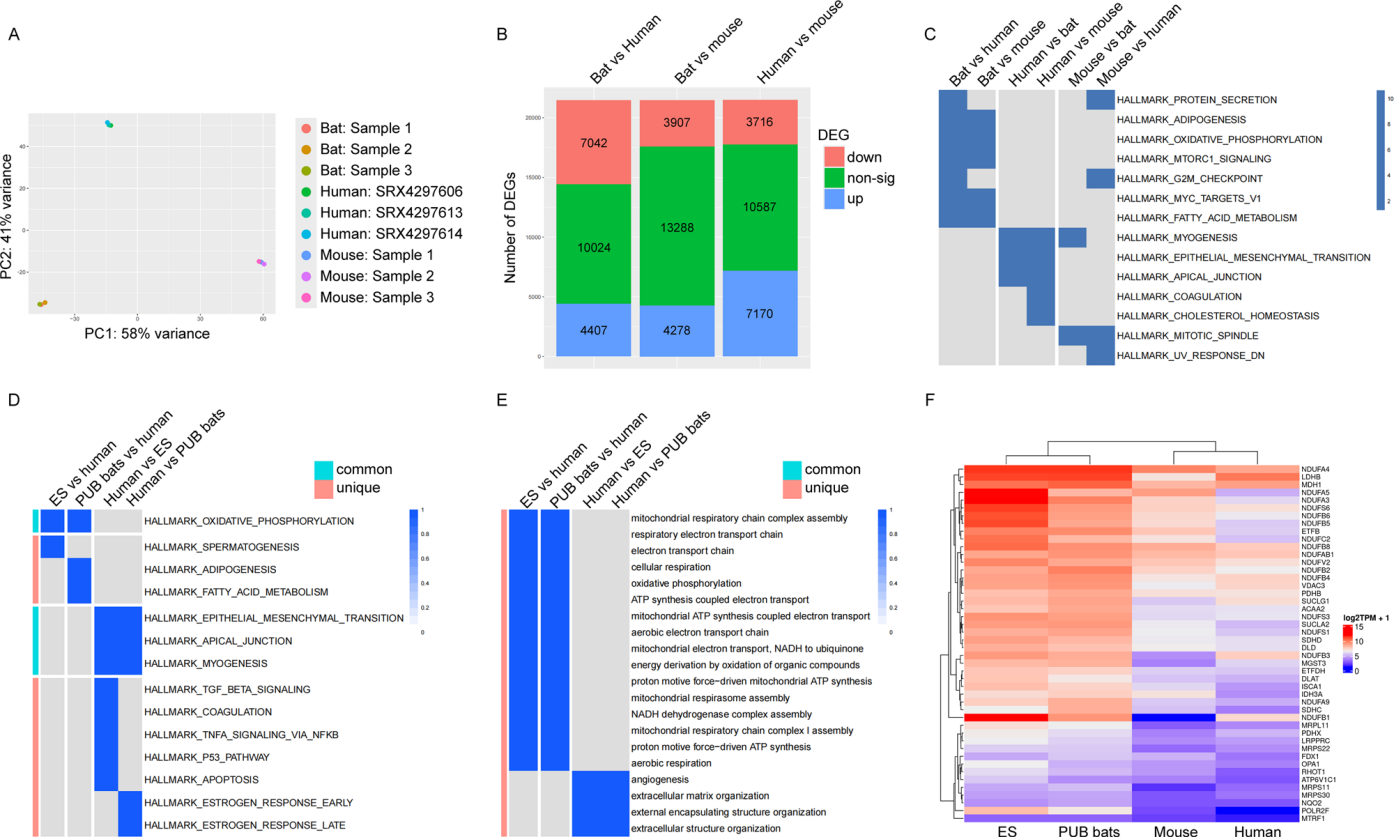
Transcriptomics and pathway analysis of bat, human, and mouse cardiac tissues. **A** Principal component analysis plot showing clustering of cardiac tissue samples by species. **B** Quantification of differentially expressed genes (DEGs) among *E. spelaea*, human, and mouse cardiac tissues, highlighting inter-species transcriptomic differences. **C** Comparison of enriched hallmark pathways in *E. spelaea*, human, and mouse cardiac tissues, showing species-specific and shared cellular processes. **D** Comparison of enriched hallmark pathways across *E. spelaea* (ES), six other bat species (PUB bats), and human cardiac tissue, showing evolutionary conserved metabolic processes in bats. **E** Comparison of enriched GO-BP pathways across seven bat species and human cardiac tissue, showing evolutionary conserved mitochondria- and energy-related processes in bats. **F** Heatmaps of DEGs in the oxidative phosphorylation hallmark pathway across seven bat species, human, and mouse cardiac tissue, showing evolutionary conserved upregulation of electron transport chain genes in bats. For (**C–E**), significant pathways in the heatmaps are represented by blue tiles, as indicated by -log10(padj). In (**D** and **E**), common pathways refer to those enriched in more than one comparison, while unique pathways are those enriched in only one comparison. Abbreviations: PUB publicly available.

We first aimed to determine whether bat hearts exhibit genetic features distinct from mouse and human hearts that underpin their cardiac adaptations. To address this, we profiled cardiac gene expression in the cave nectar bat (*Eonycteris spelaea*) using bulk tissue RNA sequencing and compared the resulting transcriptomic signatures with those of mouse and human hearts. Principal component analysis (PCA) revealed distinct clustering of samples from the three species, with human hearts showing greater similarity to bat hearts than to mouse hearts (Fig. 1A). Differential gene expression analysis uncovered 11,449 differentially expressed genes (DEGs) between bats and humans (7042 downregulated and 4407 upregulated), and 8185 DEGs between bats and mice (3907 downregulated and 4278 upregulated) (Fig. 1B). Moreover, pairwise analysis of enriched biological pathways demonstrated that bat hearts uniquely exhibited significant enrichment in oxidative phosphorylation, fatty acid metabolism, and adipogenesis pathways, while human hearts showed enrichment in epithelial-mesenchymal transition, myogenesis, and apical junction pathways (Fig. 1C).

To determine the broader applicability of these metabolic pathway adaptations, we analysed heart RNA-seq data from an additional 6 bat species (11 samples in total; Table 1) and compared them with 432 human left ventricle (LV) heart samples from the GTEx database. Pathway analysis using the MSigDB hallmark gene set consistently revealed upregulation of oxidative phosphorylation, fatty acid metabolism, and adipogenesis pathways across all 7 bat species compared to humans (Fig. 1D). Gene Ontology Biological Process (GO-BP) analysis further corroborated these findings, indicating significant enrichment for mitochondria- and energy-related biological processes in bat hearts (Fig. 1E). A heatmap derived from the oxidative phosphorylation hallmark pathway illustrated the upregulation of genes predominantly involved in the electron transport chain across all bat species relative to humans and mice (Fig. 1F). Collectively, these findings reveal a conserved metabolic signature across diverse bat species, suggesting fundamental evolutionary adaptations in cardiac energy metabolism likely driven by natural positive selection^4^. From this point forward, the data presented is from *E. spelaea* only and will be referred to as “bats”.

**Table 1:** Ecological and behavioural traits of bats used in this study

Species	Diet	Geography	Hibernation	Migration
*Eonycteris spelaea*	Nectar	South / Southeast Asia	No	No
*Artibeus jamaicensis*	Fruit	Central / South America	No	No
*Desmodus rotundus*	Blood	Central / South America	No	No
*Molossus molossus*	Insect	Central / South America	No	No
*Myotis myotis*	Insect	Europe	Yes	No
*Phyllostomus hastatus*	Generalist	Central / South America	No	No
*Rousettus aegyptiacus*	Fruit	Africa, Middle East	No	Yes

### Bat hearts exhibit a distinct metabolic profile with enhanced metabolic capacity

**Fig. 2:**
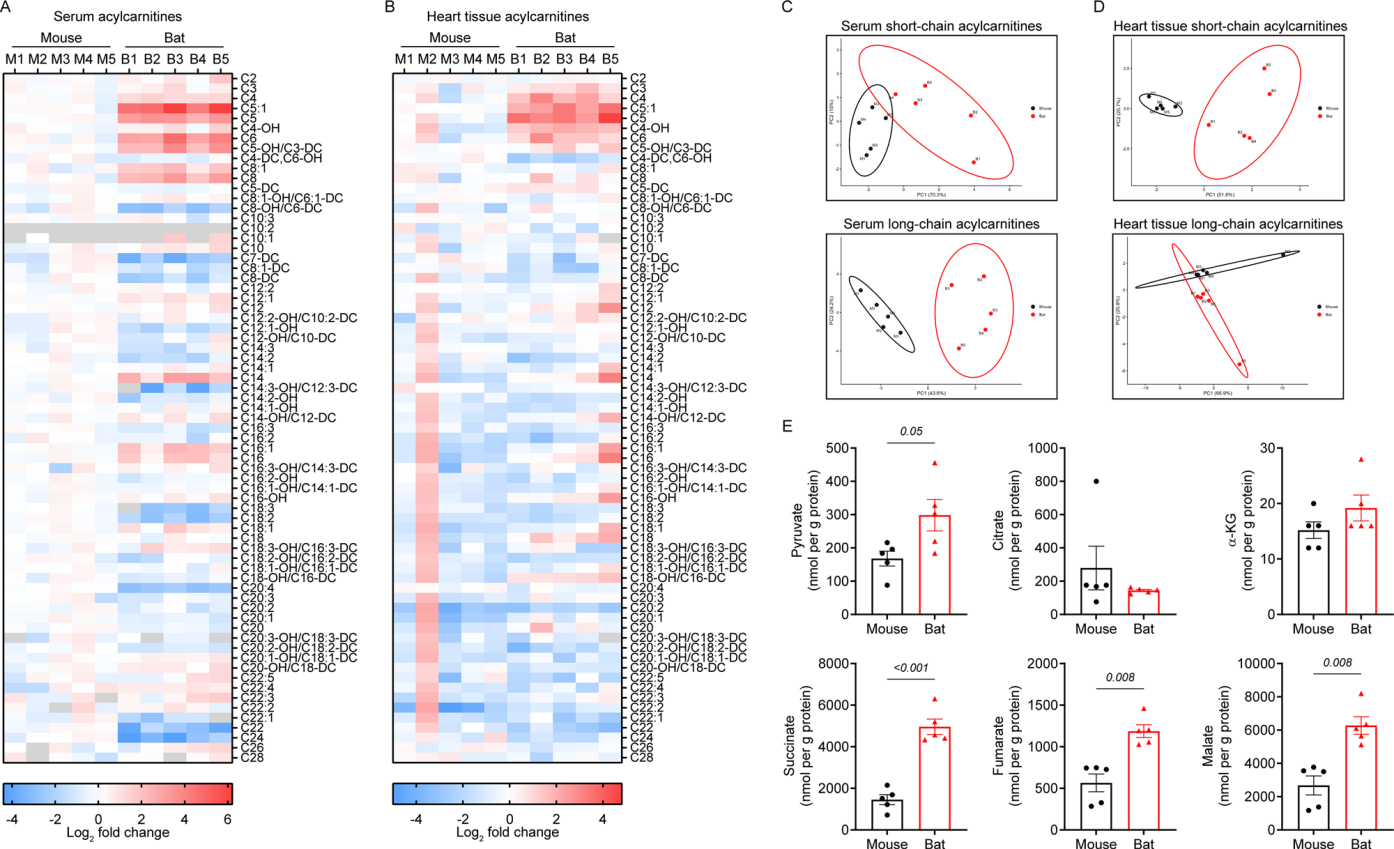
Metabolomics profiling of serum and cardiac tissues in bats and mice. **A** Heatmap of serum acylcarnitine profiles. **B** Heatmap of cardiac tissue acylcarnitine profiles. **C** Principal component analysis (PCA) plot of serum short- and long-chain acylcarnitines, showing species-specific clustering. **D** PCA plot of cardiac tissue short- and long-chain acylcarnitines, revealing species separation for short-chains only. **E** TCA cycle intermediates showing increased levels of pyruvate, succinate, fumarate, and malate in bat hearts compared to mouse hearts. Data are mean ± SEM (*n* = 5 animals per group). Welch’s t-test (**E** for pyruvate and succinate); Mann-Whitney U test (**E** for fumarate and malate).

To functionally validate the metabolic pathways observed via transcriptomic analysis, we performed targeted metabolomics on bat and mouse serum and cardiac tissues, focussing on acylcarnitine metabolites - key indicators of cardiac fuel selection^9–11^. Heatmaps revealed differential acylcarnitine profiles in bat serum (Fig. 2A), with PCA showing clear separation of short- and long-chain species from mice (Fig. 2C). Cardiac tissues also displayed distinct acylcarnitine profiles in bats (Fig. 2B), though PCA separated only short-chain species (Fig. 2D). In addition, bat hearts exhibited elevated tricarboxylic acid (TCA) cycle intermediates including pyruvate, succinate, fumarate, and malate (Fig. 2E), indicating an expanded TCA pool to fuel oxidative phosphorylation and exercise capacity^12^.

**Fig. 3:**
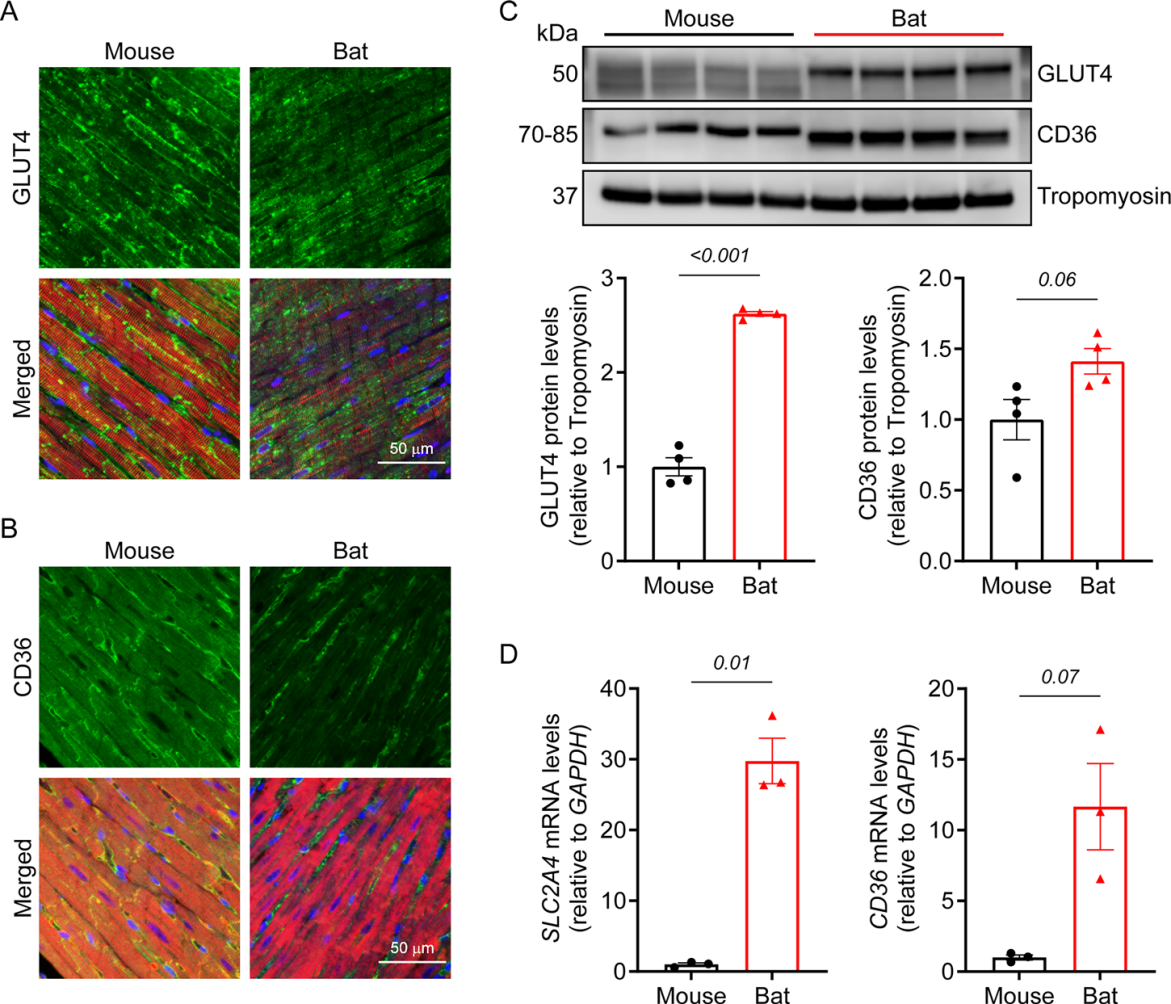
Expression of metabolic transporters in bat and mouse hearts. **A** Immunofluorescence images showing GLUT4 expression in bat and mouse cardiac tissue, counterstained with α-actinin (red) and DAPI (blue). **B** Immunofluorescence images showing CD36 expression in bat and mouse cardiac tissue, counterstained with cardiac troponin T (cTnT; red) and DAPI (blue). **C** Western blot analysis showing increased GLUT4 and CD36 protein levels in bat hearts compared to mouse hearts. **D** Real-time PCR analysis showing increased *SLC2A4* and *CD36* mRNA levels in bat hearts compared to mouse hearts. Data are mean ± SEM (*n* = 3-4 animals per group; Welch’s t-test).

Given that bat flight muscles support both carbohydrate and fatty acid β-oxidation^13,14^, we examined the expression of these substrate transporters in cardiac tissues. Immunohistochemical staining revealed that both GLUT4 (glucose transporter type 4) and CD36 (cluster of differentiation 36) were expressed in cardiac tissues of bats and mice (Fig. 3A, B, Supplementary Fig. 1A, B). Moreover, western blot analysis confirmed higher levels of both GLUT4 and CD36 in bat hearts compared to mouse hearts (Fig. 3C, Supplementary Fig. 2), consistent with greater metabolic flexibility. Although bats and mice show relatively high sequence similarity for both transporters (93% for GLUT4; 82% for CD36) (Supplementary Fig. 3), we interpreted these findings cautiously, as antibodies in cross-species comparisons are influenced by their affinity for target proteins. To corroborate the western blot data, qPCR was performed to quantify *SLC2A4* (encoding GLUT4) and *CD36* expression, which revealed an approximate 30-fold and 12-fold increase, respectively, in bat hearts compared to mouse hearts (Fig. 3D). Collectively, these findings reveal a unique cardiac fuel utilisation strategy in bats, characterised by a distinct metabolic profile and enhanced capacity, which likely enable bats to efficiently meet the fluctuating energy demands associated with flight and their unique ecological niches^13,15,16^.

### Bat hearts exhibit signs of adaptive remodelling and stress

**Fig. 4:**
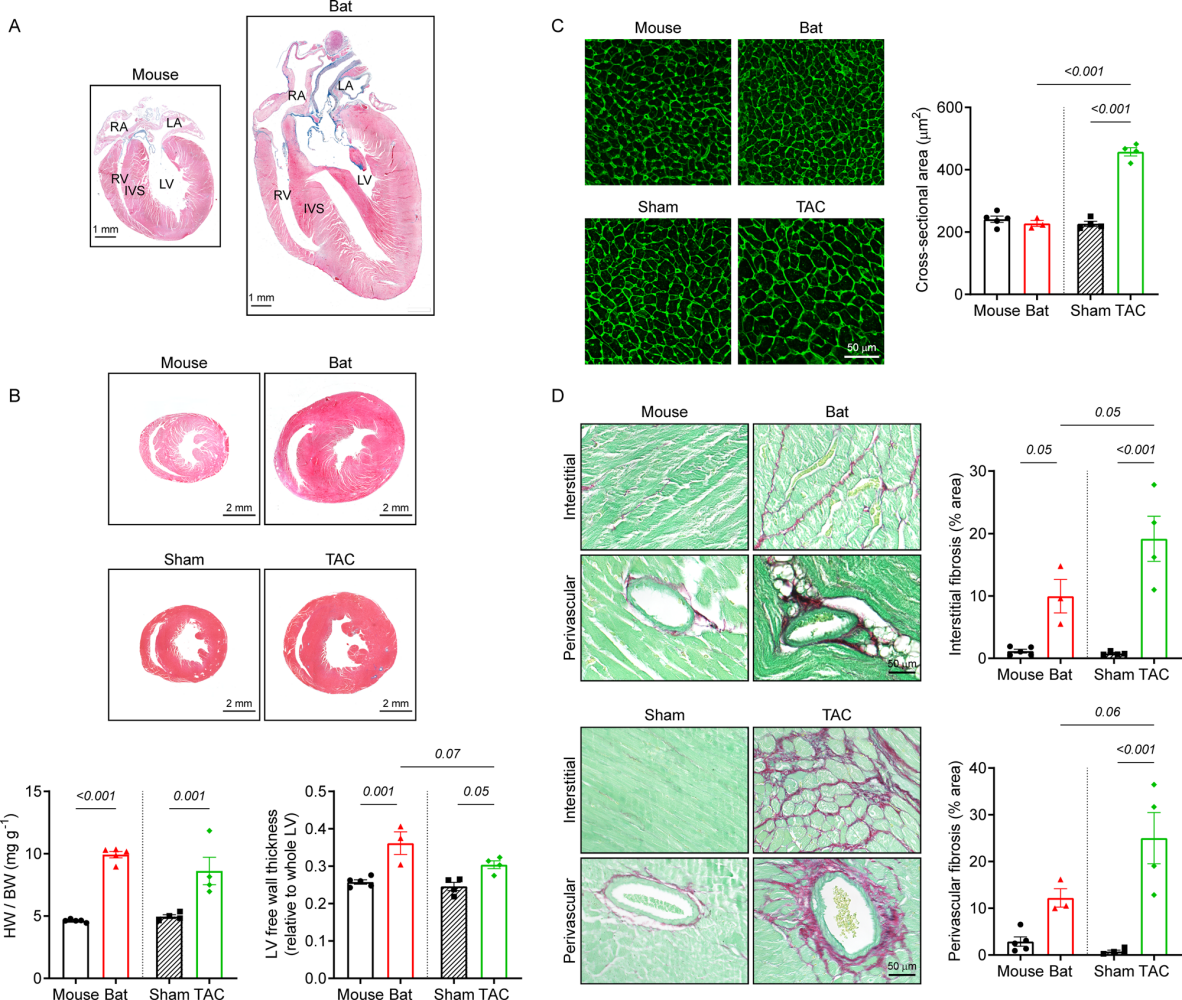
Structural comparison of adult bat, healthy mouse, and TAC mouse hearts. **A** Anatomical comparison of mouse and bat hearts. **B** Cardiac tissue cross-sections stained with Gömöri trichrome, showing that bats and TAC mice have larger hearts with thickened LV walls compared to healthy mice. **C** Cardiac tissue sections stained with wheat germ agglutinin, showing cardiomyocyte hypertrophy exclusively in the TAC mouse heart. **D** Cardiac tissue sections stained with Sirius Red/Fast Green, showing increased interstitial and perivascular fibrosis in TAC mouse hearts and to a lesser extent in bat hearts. Data are mean ± SEM (*n* = 3–5 animals per group; One-way ANOVA with Tukey post hoc test). Abbreviations: RA right atrium, LA left atrium, RV right ventricle, LV left ventricle, IVS interventricular septum.

We next investigated whether these transcriptional and metabolic adaptations were accompanied by changes in cardiac architecture. Morphometric analysis revealed striking differences between bat and mouse hearts, with the former showing notable similarities to human hearts. Longitudinal bisection from apex to base showed that bat hearts possess well-developed atrial chambers, similar to those observed in humans^17^, and in contrast to the rudimentary atrial chambers observed in mice (Fig. 4A). Moreover, bat ventricles exhibited a more elongated shape, akin to human hearts, compared to the ellipsoidal shape of mouse ventricles. Further analysis demonstrated that bats have a significantly larger relative heart size compared to healthy mice (bat vs mouse; 9.9 ± 0.3 mg g^-1^ vs 4.6 ± 0.1 mg g^-1^), and that this size is comparable to hypertrophied mice induced by transverse aortic constriction (TAC; 8.6 ± 1.1 mg g^-1^). LV free walls were strikingly thicker in bat hearts than in healthy mice, slightly exceeding TAC mice (Fig. 4B). Despite having larger hearts, cardiomyocyte size was comparable between species (Fig. 4C), suggesting the absence of pathological cellular hypertrophy in bats^18^. In contrast, TAC mice exhibited cellular hypertrophy with enlarged cardiomyocytes (Fig. 4C). Although bat hearts lack pathological hypertrophy, an increase in perivascular and interstitial fibrosis suggests the occurrence of stress (Fig. 4D). As expected, TAC mouse hearts showed greater amounts of both fibrosis types (Fig. 4D).

**Fig. 5:**
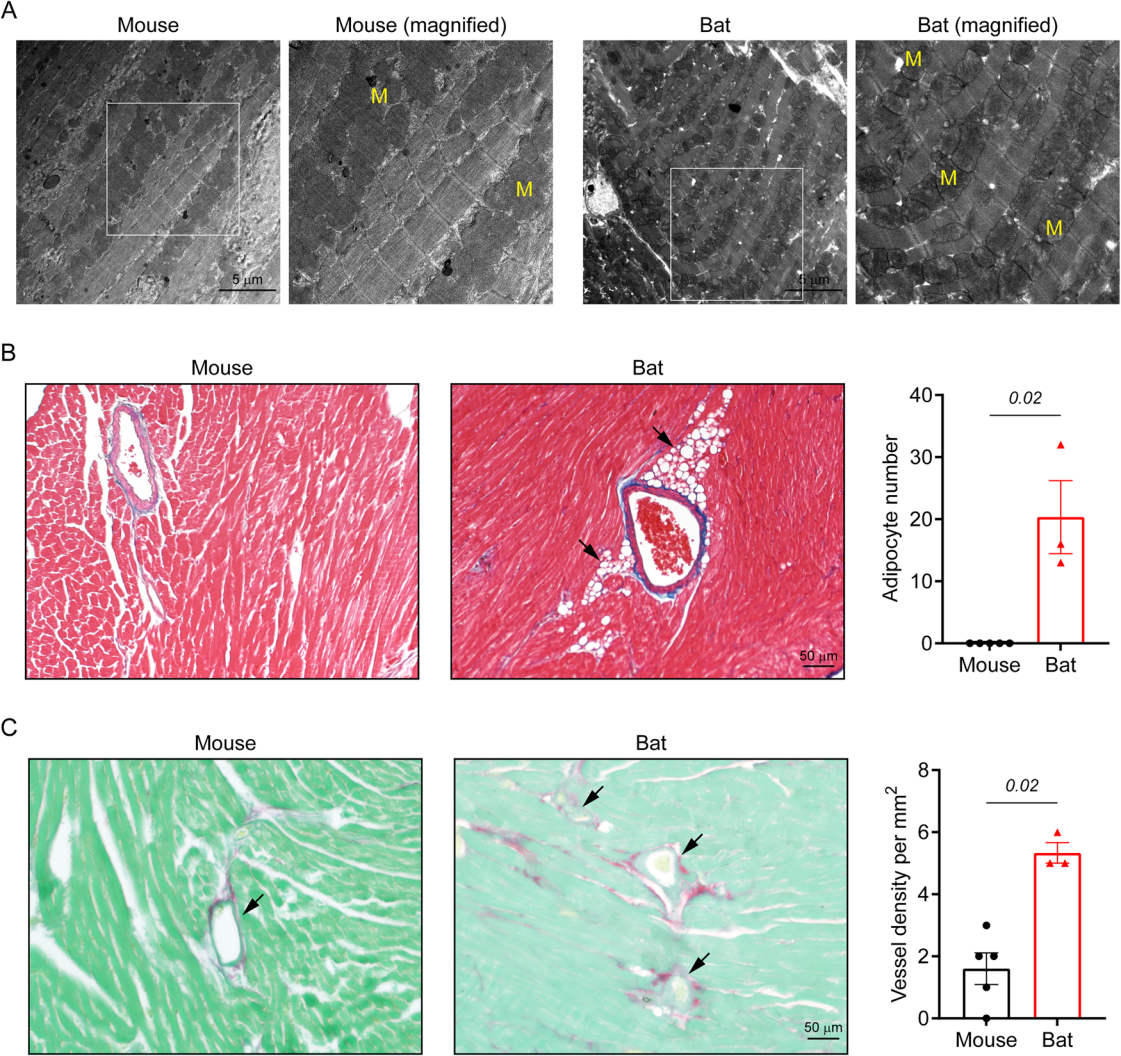
Ultrastructural and vascular characteristics of bat and mouse hearts. **A** Transmission electron microscopy images showing denser mitochondrial networks in bat hearts compared to mouse hearts. White insets represent magnified regions with ‘M’ denoting mitochondria. **B** Cardiac tissue sections showing perivascular adipocytes (black arrows) adjacent to vessels in bat hearts, a feature absent in mouse cardiac tissue. **C** Cardiac tissue sections stained with Sirius Red/Fast Green, showing higher vascular density (black arrows) in bat hearts compared to mouse hearts. Data are mean ± SEM (*n* = 3-5 animals per group; Mann-Whitney U test).

Beyond gross anatomy, we examined ultrastructural components in bat hearts that may facilitate their enhanced metabolic capacity. Transmission electron microscopy revealed more densely packed mitochondrial networks in bat hearts compared to mouse hearts (Fig. 5A). In addition, perivascular adipocytes adjacent to microvasculature and larger vessels appeared in bat hearts but were absent in mouse hearts (Fig. 5B), aligning with the enrichment of adipogenesis pathways identified in our transcriptomics analysis. Moreover, bat hearts contained a greater number of vessels per area compared to mouse hearts (bat vs mouse; 5.3 ± 0.3 per mm^2^ vs 1.6 ± 0.5 per mm^2^; Fig. 5C). Collectively, these structural characteristics suggest a sophisticated adaptive remodelling mechanism that differs fundamentally from traditional mammalian cardiac pathophysiology, while the ultrastructural features likely sustain enhanced metabolic capacity to preserve cardiac function under stress.

### Bat hearts reveal superior cardiac reserve and stress responsiveness

**Fig. 6:**
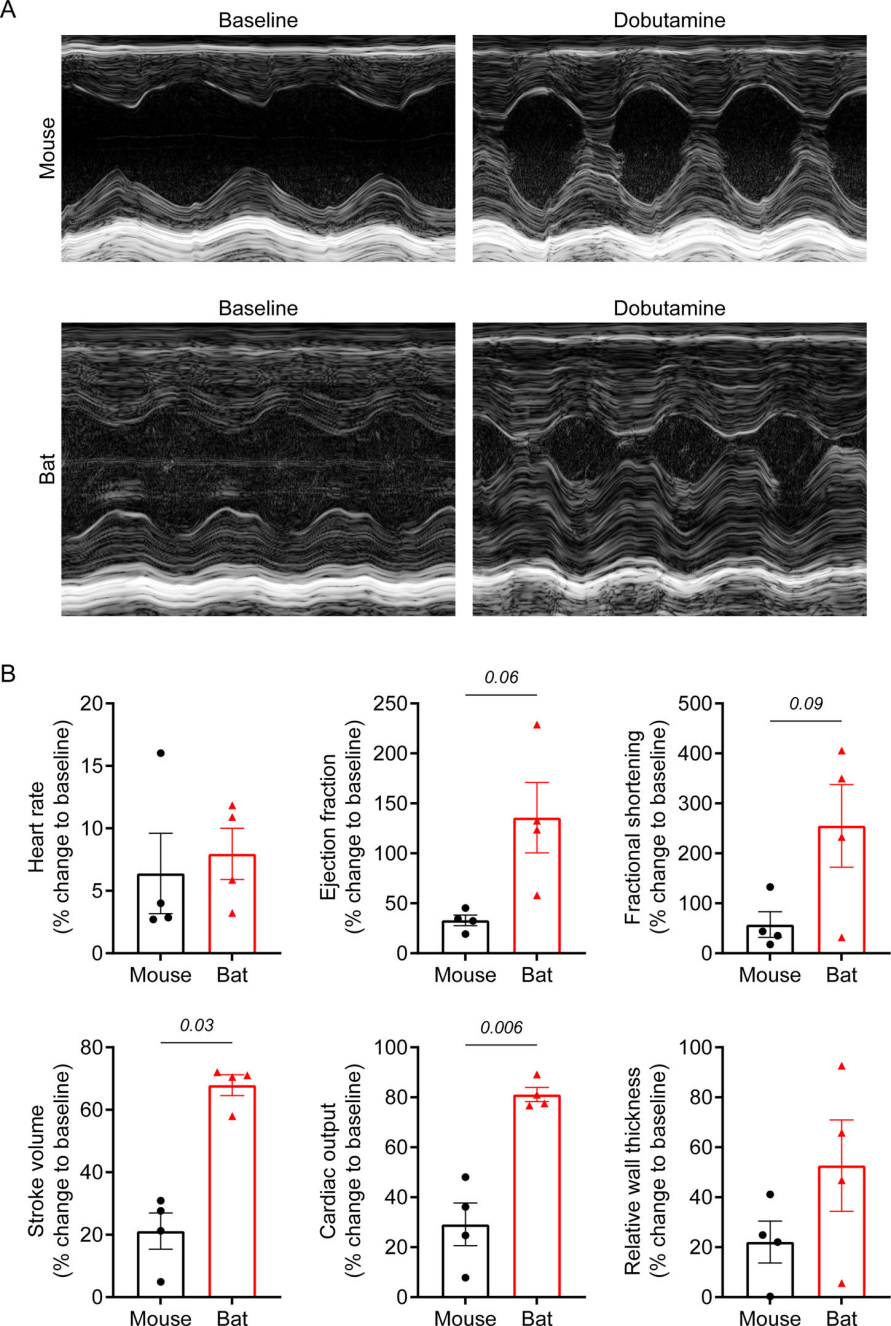
Cardiac response to dobutamine in bats and mice. **A** Representative M-mode echocardiography images of bat and mouse hearts at baseline and after dobutamine injection. **B** Effects of dobutamine on heart rate, ejection fraction, fractional shortening, stroke volume, cardiac output, and relative wall thickness in bats and mice. Data are mean ± SEM (*n* = 4 animals per groups). Welch’s t-test (**B** for ejection fraction, fractional shortening, and cardiac output); Mann-Whitney U test (**B** for stroke volume).

To evaluate whether bat hearts can sustain function under stress, we performed echocardiographic studies under baseline conditions and in response to the adrenergic agonist dobutamine. For direct comparison, bats and mice received identical isoflurane concentrations under supine positioning, with similar heart rates maintained throughout baseline echocardiography. Consistent with our morphometric findings, bats demonstrated larger LV mass and thicker LV walls compared to mice (Table 2; Fig. 6A). Despite their larger hearts, stroke volume and cardiac output remained similar between species. Notably, ejection fraction (EF) and fractional shortening (FS) were significantly lower in bats than in mice, suggesting distinct cardiac functional profiles at baseline (Table 2). To determine whether the reduced baseline EF and FS reflect impairment or adaptation, we administered a single bolus of dobutamine to assess the cardiac response in bats and mice^19^. Following dobutamine infusion, both species demonstrated similar chronotropic responses with comparable increases in heart rates (Fig. 6B). However, the inotropic response in bat hearts was substantially more pronounced than that in mice, with the former exhibiting a remarkable fold-change increase in EF (bat vs mouse; 135.8 ± 35.2% vs 32.9 ± 5.3%; Fig. 6B) and FS (bat vs mouse; 255.1 ± 82.6% vs 57.4 ± 25.7%; Fig. 6B). Moreover, stroke volume and cardiac output in bats increased nearly 3-fold, far exceeding the response observed in mice (Fig. 6B). Bat hearts also demonstrated a notable increase in relative wall thickness compared to mouse hearts, although this difference did not reach statistical significance (Fig. 6A, B). These findings suggest that bats possess superior cardiac reserve, enabling enhanced cardiac function under stress.

**Table 2:** Cardiac function of bats and mice at baseline and after dobutamine administration

	Baseline			Dobutamine		
	Mouse	Bat	*P-*value	Mouse	Bat	*P*-value
Heart rate, beats min^-1^	464 ± 12	451 ± 20	ns	493 ± 9	487 ± 26	ns
Left ventricular mass, mg	123.2 ± 4.4	433.1 ± 30.9	0.0018	134 ± 13.2	392.5 ± 53.6	0.0142
Left ventricular ejection fraction, %	62.8 ± 1.9	30 ± 4.7	0.0031	83.1 ± 1.2	66.1 ± 3.2	0.0081
Fractional shortening, %	40 ± 5.1	17.7 ± 6.2	0.057	59.2 ± 2.3	48.3 ± 1.7	0.0103
Relative wall thickness	0.51 ± 0.02	0.6 ± 0.06	ns	0.62 ± 0.02	0.89 ± 0.04	0.0024
Stroke volume, µL	46.8 ± 1.9	59.7 ± 6.7	ns	56.6 ± 2.8	99.6 ± 9.1	0.0139
Cardiac output, mL min^-1^	21.8 ± 1.4	27.3 ± 4.3	ns	27.9 ± 1.5	49.1 ± 7.1	0.0555

Data are mean ± SEM (n = 4 animals per species). Statistical significance is indicated by *P*-values; ns indicates not significant.

**Fig. 7:**
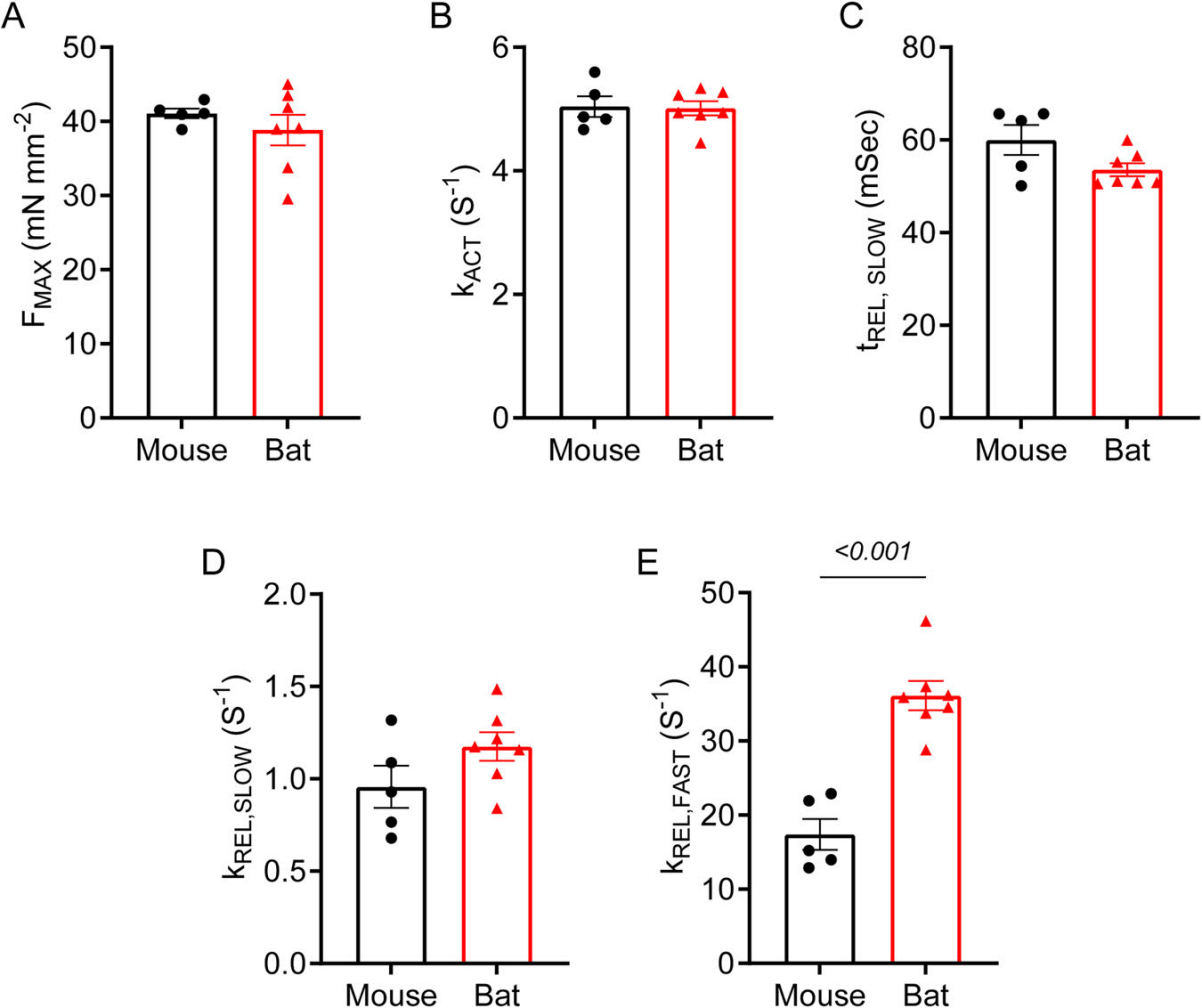
Myofibril mechanics in bat and mouse cardiomyocytes. **A** Force generation in bat and mouse myofibrils. **B** Activation kinetics, represented by the rate constant of tension development (kACT), in bat and mouse myofibrils. **C** Duration of linear phase relaxation in bat and mouse myofibrils. **D** Linear phase relaxation kinetics in bat and mouse myofibrils. **E** Exponential phase relaxation kinetics, represented by the rate constant of exponential relaxation (fast kREL), in bat and mouse myofibrils. Data are mean ± SEM (*n* = 5–7 animals per group; Welch’s t-test for **E**).

We next compared bat and mouse cardiac myofibrils to elucidate the mechanisms underlying the enhanced cardiac reserve. Although force generation and activation kinetics were similar between the two species (Fig. 7A, B), bats showed a non-significant tendency towards faster linear phase relaxation, with a slight decrease in duration and increase in rate of relaxation (Fig. 7C, D). Notably, bat myofibrils demonstrated approximately 2-fold faster exponential phase relaxation kinetics (bat vs mouse; 36.1 ± 2.0 s^-1^ vs 17.4 ± 2.1 s^-1^; Fig. 7E). This enhanced relaxation may support quicker transitions between contraction and relaxation states, particularly contributing to improved diastolic function and enabling more effective filling of the heart between contractions^20^.

### Bat cardiomyocytes exhibit resistance to Angiotensin II-induced stress

**Fig. 8:**
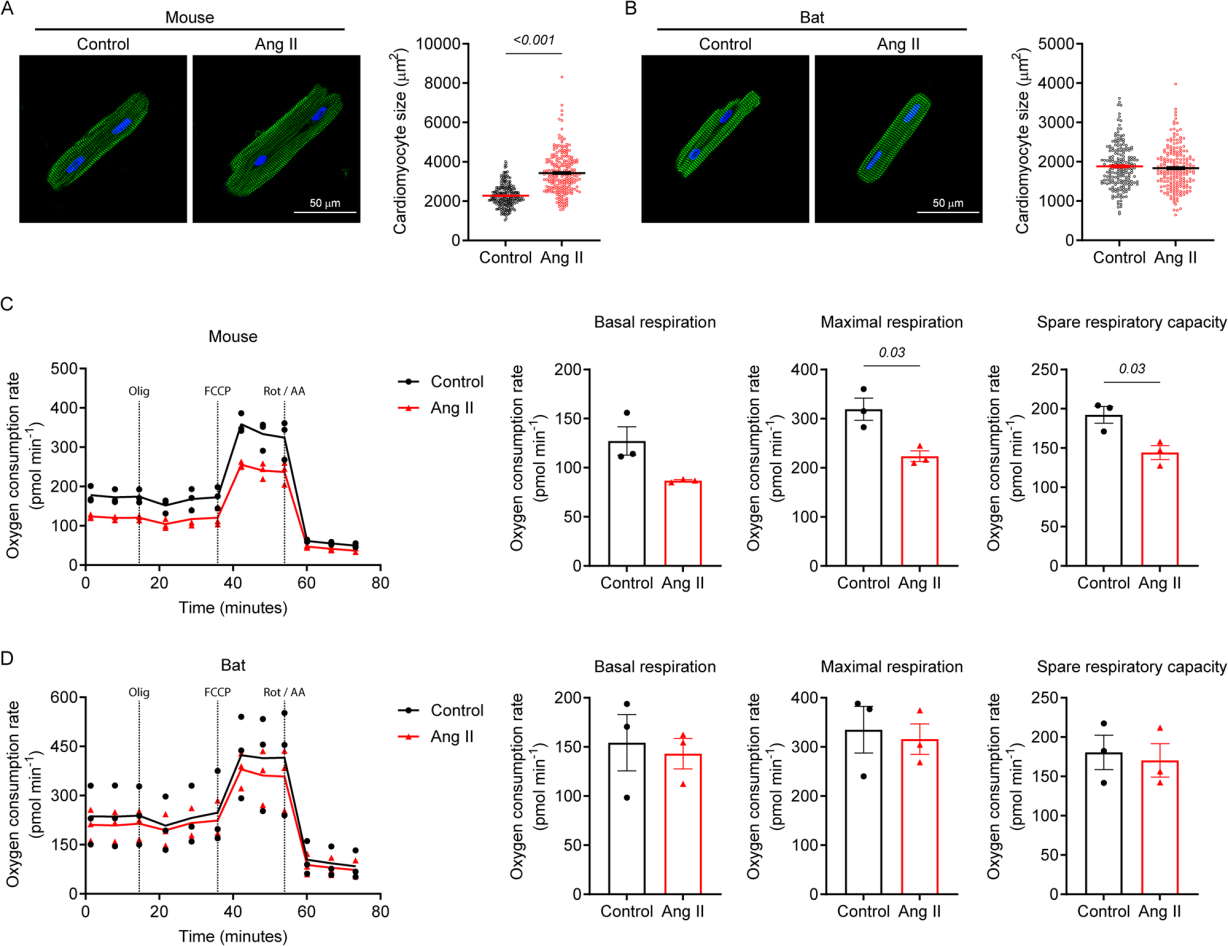
Effects of Angiotensin II on cardiomyocyte hypertrophy and mitochondrial function in bats and mice. **A** Immunofluorescence images showing Ang II induced cellular hypertrophy in mouse cardiomyocytes. **B** Immunofluorescence images showing no effect of Ang II on bat cardiomyocyte size. **C** Oxygen consumption rate trace of mouse cardiomyocytes showing Ang II-induced mitochondrial dysfunction with changes in respiratory parameters. **D** Oxygen consumption rate trace of bat cardiomyocytes showing no effect of Ang II on mitochondrial function with no changes in respiratory parameters. Data are mean ± SEM (*n* = 4 animals per group, 50–73 cells per animal for cellular hypertrophy assessment; *n* = 3 animals per group for mitochondrial function analysis). Mann-Whitney U test (**A** and **B**); Welch’s t-test (**C** for maximal respiration and spare respiratory capacity). Abbreviations: Olig oligomycin, Rot rotenone, AA antimycin A.

We speculated that the enhanced inotropic response in bats following dobutamine administration resulted from cardiometabolic adaptations that sustain myocardial energetics under stress. To evaluate whether bat hearts are truly resistant to pathological stress, ventricular cardiomyocytes isolated from bats and mice were treated for 24 h with angiotensin II - the effector molecule of the renin-angiotensin system, which has been shown to induce cardiomyocyte hypertrophy and mitochondrial dysfunction in neonatal rat cardiomyocytes^21,22^. While Ang II elicited cellular hypertrophy in mouse cardiomyocytes, it failed to do so in bat cardiomyocytes (Fig. 8A, B). Moreover, Ang II induced mitochondrial dysfunction in mouse cardiomyocytes, as evidenced by reduced basal respiration, maximal respiration, and spare reserve capacity (Fig. 8C). However, these impairments were absent in bat cardiomyocytes (Fig. 8D). Collectively, these findings affirm that bat hearts may be resistant to pathological remodelling, with preservation of myocardial energetics under stress likely resulting from integrated cardiometabolic adaptations that sustain cardiac function during the extreme energy demands of powered flight.

## Discussion

Our comprehensive investigation of bat cardiometabolic adaptations reveals several findings: (1) transcriptomic analysis across 7 bat species demonstrated enriched signatures in oxidative phosphorylation and fatty acid metabolism, suggesting a conserved evolutionary metabolic strategy; (2) metabolomics profiling indicated a distinct acylcarnitine profile with elevated TCA cycle intermediates, revealing enhanced metabolic capacity; (3) structural analysis supported a highly metabolic environment characterised by increased mitochondria, adipocyte, and vascular densities; (4) functional analysis revealed superior cardiac reserve, stress-induced performance improvements, and remarkable cardiomyocyte resistance to hypertrophic and mitochondrial stress. These findings collectively highlight the extraordinary physiological adaptations that enable bat hearts to meet the extreme energy demands of powered flight.

Our findings on the structural adaptations of *E. spelaea* corroborate and extend previous studies on bat hearts. Compared to mice, *E. spelaea* has a relatively larger heart to support the high oxygen demands of flight^5^. These hearts also showed increased vascular density, aligning with previous observations of elevated capillary density in bat hearts and skeletal muscles^23^. Ultrastructural analysis revealed densely packed mitochondrial networks in *E. spelaea* cardiomyocytes, paralleling findings in *Eidolon helvum* and *Pipistrellus pipistrellus*, which exhibit higher mitochondrial content and other specialised arrangements to promote efficient energy utilisation^6,7^. Collectively, these conserved features indicate evolutionary specialisation for enhanced aerobic metabolism and greater energy production capacity in bat hearts. In addition, we identified increased cardiac fibrosis, which may have resulted from cumulative “wear and tear” associated with prolonged inversion during roosting—a behaviour previously shown to cause cardiac injury in bats^8^. Alternatively, the increased fibrotic content may serve an adaptive role by providing additional structural support under extreme metabolic demands. Notably, despite their larger size and thicker ventricular walls, *E. spelaea* hearts showed no evidence of cardiomyocyte hypertrophy, suggesting a form of adaptive remodelling distinct from pathological enlargement^18^. Future studies comparing exercise-trained mouse hearts, which lack fibrosis, with bat hearts may help further define this unique physiological-pathological spectrum.

Transcriptomic analysis across seven bat species revealed enrichment of pathways related to fatty acid metabolism and oxidative phosphorylation compared with human and mouse hearts. This finding suggests evolutionary reinforcement of fatty acid utilisation as the primary cardiac energy source in bats. Metabolomics profiling of *E. spelaea* supported this observation, showing elevated levels of short- and long-chain acylcarnitines in serum and accumulation of short-chain acylcarnitines and TCA cycle intermediates in cardiac tissue. These patterns imply enhanced fatty acid β-oxidation and oxidative phosphorylation, with differences between serum and tissue profiles reflecting systemic versus local metabolic processes. The high short-chain acylcarnitine content in cardiac tissue may relate to the bats’ nocturnal feeding behaviour and fasting state during sampling, thereby reflecting metabolic trends seen in hibernating and aroused bats^24^. Supporting this interpretation, a recent study demonstrated that *Rousettus aegyptiacus* shifts from predominant fatty acid β-oxidation during flight to increased ketone body utilisation during low-activity and roosting states, further underscoring bats’ dynamic metabolic flexibility^25^. Different ecological niches further highlight the flexibility of bat metabolism through their ability to rapidly channel ingested nutrients into energy production despite dietary diversity across species. For example, nectarivorous species use dietary sugars during flight, while insectivorous forms oxidise protein-rich diets^15^. In *R. aegyptiacus* and *Glossophaga soricina*, up to 80% of hovering-flight energy can be derived from recently ingested sugars^13,15,16^. However, during fasting or extended flight, conserved fatty acid pathways likely sustain cardiac energy demands^3^. Supporting this, *E. spelaea* hearts contain perivascular adipocytes and show enriched adipogenesis pathways, suggesting localised energy reservoirs consistent with prior reports of cardiac fat deposits in bats^26,27^. Furthermore, elevated expression of GLUT4 and CD36 transporters indicates enhanced substrate uptake and metabolic flexibility under varying energetic conditions. Collectively, these adaptations confer remarkable metabolic flexibility and efficiency to bat hearts, enabling sustained performance during high-energy flight.

The functional analysis of *E. spelaea* hearts revealed key insights into bat cardiac physiology. At baseline, *E. spelaea* exhibited lower EF and FS compared with mice, consistent with the distinctive cardiac innervation patterns reported in other bat species^28^, and this may reflect an evolutionary adaptation for energy conservation during rest. Upon dobutamine stimulation, however, *E. spelaea* hearts exhibited a marked increase in EF, FS, stroke volume, and cardiac output, indicating a superior cardiac reserve compared with mouse hearts. These findings parallel adaptations seen in other mammalian species exposed to extreme physiological demands, such as the cyclic bradycardic state in *Uroderma bilobatum*^3^ and the cardiac plasticity of naked mole-rats^29^. Mechanistically, this enhanced cardiac reserve may be underpinned by differences in β-adrenergic sensitivity, calcium handling, and metabolic flexibility. Our myofibril data revealed approximately 2-fold faster exponential phase relaxation kinetics in *E. spelaea* cardiomyocytes, potentially reflecting superior calcium handling similar to that observed in the superfast muscles of echolocating bats, where rapid calcium transients and increased fibre shortening velocities are essential for extreme physiological performance^30^. Moreover, a recent study showed that bats express an N-terminally truncated isoform of cardiac troponin I through alternative exon 3 splicing^31^, potentially reducing myofilament calcium sensitivity and enabling faster myocardial relaxation and diastolic filling. The combination of superior cardiac reserve and enhanced myofibrillar relaxation provides compelling evidence of evolved mechanisms enabling bats to sustain intense metabolic demands.

Our investigation into the response of *E. spelaea* cardiomyocytes to Ang II revealed a notable resistance to pathological alterations associated with cellular hypertrophy and mitochondrial dysfunction^21^, a finding that is particularly striking given that Ang II is well known to induce cardiac hypertrophy through mitochondrial reactive oxygen species (ROS) generation. To explain this phenomenon, we first considered whether the observed resistance might arise from incompatibility between the human-derived Ang II ligand used in our culture system and the bat angiotensin II type 1 receptor (AT1R). However, protein sequence analysis revealed functionally conserved AT1R domains across species, with bat-human and bat-mouse comparisons each displaying 97% similarity (98% for mouse-human AT1R), thereby ruling out ligand-receptor incompatibility as a plausible cause. Alternatively, we hypothesised that unique downstream mechanisms in bat cardiomyocytes that mitigate ROS generation underlie this resistance, consistent with prior studies on bat physiology and longevity. For instance, mitochondria from *Myotis lucifugus* generate significantly less hydrogen peroxide per unit of oxygen consumed than those of shorter-lived, non-flying mammals, supporting the free radical theory of aging^32^. Moreover, several South American bat species exhibit exceptionally high antioxidant defences, including elevated superoxide dismutase and catalase activities, along with abundant α-tocopherol and β-carotene in various tissues^33^. This enhanced antioxidant capacity, together with the ability to modulate defence systems during torpor-activity transitions, likely contributes to bats’ unique ecophysiological adaptations and longevity^33^. Collectively, these findings suggest that the resistance of *E. spelaea* cardiomyocytes to Ang II-induced pathological changes may arise from evolved mechanisms that mitigate ROS generation and preserve cellular homeostasis, thereby supporting their capacity to withstand the extreme metabolic demands of flight while maintaining extended longevity. Further elucidation of these stress‑resistance mechanisms holds broad implications for understanding cardiac resilience across species and may provide new perspectives on evolved cardioprotective strategies in mammals.

Our study, while providing key insights into bat cardiometabolic adaptations, is constrained by several key limitations. First, comparing *E. spelaea* with mice entails the phylogenetic and ecological non-independence inherent to two-species comparisons^34^. Differences in cardiac metabolism, structure, and stress responses may reflect not only adaptive evolution to flight but also lineage-specific history and allometric effects. Without a broader phylogenetic framework, it is difficult to disentangle adaptive mechanisms from inherited or size-related traits. Expanding comparative analyses across multiple mammalian species with varying metabolic and ecological profiles would better capture adaptive trajectories and allow more rigorous evolutionary inference. Second, fundamental dietary differences persist between laboratory mice (*ad libitum* chow) and captive *E. spelaea* (commercial nectar feed). While captivity standardises bat nutrition compared to wild variability, nectivorous diets remain inherently sugar-rich and episodic, influencing metabolic substrate utilisation and energy storage. These contrasts emphasise the need for caution when attributing physiological differences purely to evolutionary adaptation rather than environmental or dietary context^34^. Third, only *E. spelaea* and male specimens were examined for most studies. Restricting analyses to a single bat species limits generalisability across the phylogenetic and ecological diversity of Chiroptera, while exclusive use of males precludes assessment of potential sex-linked differences in cardiac physiology and metabolic regulation. Including females and additional bat species in future work would provide a more comprehensive understanding of interspecies and sex-related variation in cardiac adaptation. Finally, mechanistic uncertainties persist regarding the molecular pathways underlying cardiomyocyte stress resistance, and our in vitro experiments may not fully capture the complex physiological demands of active flight. Together, these limitations highlight the need for broader, integrative studies to fully elucidate the molecular and physiological basis of bats’ extraordinary cardiometabolic adaptations.

In conclusion, our investigation reveals that bat hearts exhibit unique cardiometabolic adaptations that support extreme physiological performance during powered flight. These include enhanced metabolic capacity, distinct structural characteristics, superior cardiac reserve, and notable stress resistance. By elucidating bats’ sophisticated cardiac biology, this study offers critical insights into metabolic efficiency and resilience, advancing our understanding of cardioprotection and evolutionary biology.

## Methods

### Animal models and surgical procedures

We have complied with all relevant ethical regulations for animal use. All experiments were approved by the SingHealth Institutional Animal Care and Use Committee (IACUC; protocol numbers 2023/SHS/1847, 2015/SHS/1088, and 2020/SHS/1582). Young adult male C57BL/6 J mice (10–12 weeks old; body weight 26.7 ± 0.6 g) were obtained from inVivos, Jackson Laboratory, Singapore. Young adult male bats (*Eonycteris spelaea*, 18–24 months old; body weight 63.6 ± 1.3 g) were sourced from a local captive-breeding colony housed at the National Large Animal Research Facility (NLARF) in Singapore^35^. Mice and bats were selected at ages reflecting sexual maturity and completion of rapid early growth (which can extend up to 14 months post-birth in bats^35^), rendering their physiological ages comparable despite chronological differences.

For transverse aortic constriction (TAC) surgery, 10-week-old mice were intubated under general anaesthesia induced by intraperitoneal injection of 100 mg kg^-1^ ketamine and 10 mg kg^-1^ xylazine, with anaesthesia maintained using 2% isoflurane in oxygen. Ventilation was provided using a rodent ventilator. A left lateral thoracotomy was performed to expose the aortic arch, and the ascending aorta (between the brachiocephalic and left carotid arteries) was ligated with a silk suture using a 27 G needle as a spacer, which was promptly removed after ligation. Control animals (referred to as healthy mice) underwent sham operations without aortic ligation. Mice were monitored postoperatively for behaviours changes indicative of pain or distress. Pre-operative care included analgesia (intraperitoneal injection of 0.1 mg kg^-1^ buprenorphine) and antibiotics (Baytril, 5 mg kg^-1^ in drinking water). Cardiac remodelling was assessed 8 weeks after surgery. At the terminal timepoint, mice were euthanised by intraperitoneal injection of 100 mg kg^-1^ ketamine and 10 mg kg^-1^ xylazine, while bats received an intraperitoneal injection of 100 mg kg^-1^ pentobarbital.

### RNA sequencing and bioinformatics

RNA was extracted from frozen bat and mouse cardiac tissues (*n* = 3 animals per group) using the RNeasy Plus Micro Kit (Qiagen). RNA sequencing libraries were prepared with Illumina TruSeq stranded mRNA and Nugen Ovation amplification kits, followed by sequencing on a HiSeq 4000 platform (Novogene Technology CO., Ltd, Singapore). Healthy human heart RNA-Seq data from three individuals were obtained from GSE116250 (SRX4297606, SRX4297613, SRX4297614). Fastq reads were aligned using the nf-core rnaseq pipeline (3.14.0) with the star_salmon workflow. Length-scaled counts were generated for each sample and differential gene expression analysis was performed using DESeq2 (padj < 0.05 and abs(logFC) > 1).

For the analysis of publicly available bat heart RNA-Seq data, fastq reads were downloaded for *Artibeus jamaicensis* (SRR13417529, SRR13417530, SRR13417531, SRR13417532), *Desmodus rotundus* (SRR13417566), *Molossus molossus* (SRR13417562), *Myotis myotis* (SRR11528219, SRR11528220), *Phyllostomus hastatus* (SRR13417565) and *Rousettus aegyptiacus* (SRR2913354, SRR2914359). Since raw fastq data was not readily available for human GTEx left ventricle (LV) samples, publicly deposited TPM data for human GTEx (V8) was downloaded for analysis. A complete TPM matrix included 432 human GTEx LV samples, three *E. spelaea* heart samples, three mouse heart samples, and 11 publicly available bat samples from six different species. 15,230 genes that were annotated in all samples were used for differential gene expression analysis using Wilcoxon tests^36^ and significant DEGs were identified using the criterion: abs(logFC) > 1 and FDR < 0.01. Pathway analyses of significant DEGs were performed against the MSigDB hallmark and GO-BP gene sets using the genekitr ORA (over-representation test) function^37^.

### Metabolomics

Acylcarnitine and organic acid (TCA cycle intermediates) analyses were performed at the Duke-NUS Metabolomics Facility. Cardiac tissues were weighed and homogenised in ice-cold 50% acetonitrile containing 0.3% formic acid at a concentration of 50 mg tissue per mL of homogenate. Organic acids were extracted from the tissue homogenate and derivatised to their trimethylsilyl forms before quantitation by gas chromatography-mass spectrometry (GC-MS). Acylcarnitines and amino acids were extracted and derivatised to their methyl and butyl esters, respectively, then quantitated using liquid chromatography-mass spectrometry (LC-MS). The results were normalised to tissue weight to account for variations in sample input.

### Western blotting

Western blots were performed using a previously described protocol^38^. Proteins (25 μg) extracted from cardiac tissues were separated on 4–12% Bis-Tris Bolt Gels (Thermo Fisher Scientific, MA, USA) and transferred to nitrocellulose (NC) membranes using the iBlot Dry Blotting System (Thermo Fisher Scientific). NC membranes were blocked and incubated overnight with primary antibodies: CD36 (1:500; #ab64014, Abcam, Cambridge, UK) and GLUT4 (1:1000; #2213, Cell Signaling Technology, MA, USA). The following day, membranes were washed, incubated with HRP-conjugated secondary antibodies, and developed using SignalFire™ ECL Reagent (Cell Signaling Technology). Tropomyosin (1:20,000, #3910, Cell Signaling Technology) was used as a loading control. Blots were imaged using the C-DiGit Blot Scanner (LI-COR, NE, USA) and densitometry was performed using Image Studio software (LI-COR).

### Real-time PCR

RNA was isolated from cardiac tissues and converted to cDNA using SuperScript III First-Stran Synthesis System (Thermo Fisher Scientific) as previously described^39^. cDNA templates were amplified using QuantiFast SYBR Green PCR Kit (Qiagen, Hilden, Germany) on a Rotor-Gene Q (Qiagen) with the following cycling conditions: 5 min at 95 °C, followed by 40 cycles of 10 s at 95 °C and 30 s at 60 °C. Relative quantification was performed using the ^ΔΔ^Ct method and normalised to *GAPDH*. Primer sequences are listed in Supplementary Table 1.

### Transmission electron microscopy

Left ventricular tissues from mice and bats were processed for transmission electron microscopy (TEM) using a standardised protocol. Tissue fragments were fixed in 0.1 M phosphate buffer (2% paraformaldehyde, 2% glutaraldehyde) at 4 °C, washed, and post-fixed in 2% osmium tetroxide with potassium ferrocyanide for 2 h at room temperature. After dehydration through an ethanol series, tissues were embedded in resin and ultrathin sectioned at 90 nm using an EM UC7 ultramicrotome (Leica Microsystems, Wetzlar, Germany). Ultrastructural imaging was performed on a FEI Tecnai Spirit G2 TEM (FEI Company, OR, USA) with an FEI Eagle 4k digital camera at 2000x magnification.

### Histology and immunofluorescence

Cardiac tissues were harvested, washed with ice-cold phosphate-buffered saline, and immediately fixed in 10% neutral buffered formalin. After processing and paraffin embedding, tissues were sectioned at 4 µm thickness (Leica AG, Solms, Germany). Tissue sections were deparaffinised using Histo-Clear and rehydrated through a graded ethanol series (100 to 70%). For morphological analysis, Gömöri trichrome staining was performed. Fibrosis was assessed using a Sirius Red and Fast Green kit (#9046, Chondrex, WA, USA). Images were acquired using a Zeiss Axiovert 200 M microscope (Carl Zeiss, Oberkochen, Germany), and the fibrotic area was quantified as a percentage of the total image area using ImageJ software, with five sections analysed per sample.

For immunofluorescence, antigen retrieval was performed using Bull’s Eye Solution (#BULL1000MX, Biocare Medical, CA, USA) at 98 °C for 10 min. Tissue sections were blocked with 5% BSA for 45 min, then incubated with WGA Alexa Fluor™ 488 Conjugate (1:200, #W11261, Thermo Fisher Scientific, MA, USA) for 3 hours at room temperature. Tissues were mounted using TrueVIEW with DAPI (#SP-8500-15, Vector Laboratories, CA, USA). Images for cardiomyocyte cross-sectional area analysis were acquired using a fluorescence microscope (Carl Zeiss), with approximately 100 cells measured per sample. For CD36 and GLUT4 immunostaining, antigen retrieval was performed using citrate buffer (#ab64214, Abcam, Cambridge, UK) at 98 °C for 10 min. Tissue sections were blocked with 5% BSA for 1 hr and incubated overnight with primary antibodies: CD36 (1:200; #ab64014, Abcam), GLUT4 (1:200; #2213, Cell Signalling Technologies), cardiac Troponin T (1:200, #ab10214, Abcam), and α-actinin (1:200; #ab137346, Abcam). The following day, sections were washed and incubated with Alexa Fluor^TM^ 488 (#A-11070) and Alexa Fluor^TM^ 555 (#A-21422) secondary antibodies (both from Thermo Fisher Scientific). Tissues were mounted using TrueVIEW with DAPI (#SP-8500-15, Vector Laboratories).

To measure isolated cardiomyocyte size, cells were fixed with 4% paraformaldehyde, permeabilised with 0.3% Triton X-100, blocked with 5% BSA, and incubated overnight with anti-sarcomeric α-actinin primary antibodies (#ab137346, Abcam). Cells were then probed with Alexa Fluor™ Plus 488 secondary antibodies (#A-11070, Thermo Fisher Scientific) and counterstained with DAPI. Representative images of isolated cardiomyocytes were captured using an inverted confocal microscope (LSM710, Carl Zeiss). All image analyses were performed using ImageJ software.

### Echocardiography

Echocardiography was performed using a Vevo 2100 system (Visual Sonics Inc., Toronto, Canada) equipped with a 38 MHz MicroScan transducer (frequency range 18–38 MHz) at the SingHealth Experimental Medicine Centre (SEMC). Anaesthesia was induced with 5% isoflurane and maintained at 1% isoflurane throughout the procedure. A comprehensive examination included two-dimensional (2D) B-mode imaging, motion-mode (M-mode) imaging, and tissue Doppler imaging (TDI). Images were acquired at baseline and following an intraperitoneal bolus injection of dobutamine (10 μg g^-1^ body weight), using the same anaesthesia protocol throughout to ensure comparability between measurements. To directly compare dobutamine-induced changes between mice and bats, percentage change from baseline was calculated for each echocardiographic parameter within individual animals, then these individual within-animal change scores were compared between species using simple two-group tests.

### Myofibril mechanics assay

Myofibril mechanics were quantified as previously described^40^. Briefly, the fast solution switching technique was used to assess myofibril contractile properties. Left ventricular (LV) sections were skinned overnight in rigor solution (132 mM NaCl, 5 mM KCl, 1 mM MgCl_2_, 10 mM Tris, 5 mM EGTA, pH 7.1) containing 0.5% Triton X-100, protease inhibitors (10 μM leupeptin, 5 μM pepstatin, 200 μM PMSF, and 10 μM E64), 500 μM NaN_3_, and 500 μM DTT at 4 °C. Skinned LVs were homogenised in relaxing solution (pCa 9.0, where pCa = -log10[calcium concentration]). Myofibril suspensions were transferred to a temperature-controlled chamber (15 °C) and mounted between two microtools: one connected to a motor for length changes (Mad City Labs), the other a calibrated cantilevered force probe (12.2 μm μN^-1^; frequency response 2-5 kHz). Myofibril length was set at approximately 2.2 μm. Sarcomere lengths and myofibril diameters were measured using ImageJ software (NIH). Myofibrils were activated and relaxed by rapid translation between solutions of different pCa. Mechanical and kinetic parameters measured included resting tension, maximal tension, rate constant of tension development (kACT), duration of linear relaxation, and rate constant of exponential relaxation (fast kREL). At least one sample from each group was analysed daily, with all cultured cells in an experiment harvested on the same day. While experiments were not blinded during conduct, data analysis was performed in a blinded fashion.

### Cardiomyocyte isolation and culture

Cardiomyocytes from mice and bats were isolated using a Langendorff-free direct needle perfusion method^41^. Animals were anaesthetised with 75% CO_2_/25% O_2_ at 1 L min^-1^ flow rate containing isoflurane, maintained at 2% via nose cone. The heart was flushed by injecting EDTA buffer into the right ventricle, then excised and transferred to fresh EDTA buffer. Cardiac tissues were digested with collagenase buffer until softened. Ventricles were separated, and the tissue was gently dissociated by pipetting. The cell suspension was filtered through a 100 µm filter, and cardiomyocytes were collected by gravity sedimentation for 20 minutes, followed by three additional sedimentations in calcium reintroduction buffers. Yield and viability were assessed using a haemocytometer. For culture, cardiomyocytes were suspended in M199 medium (#M4530, Merck, Darmstadt, Germany) supplemented with 5% FBS, 10 mmol L^-1^ BDM (2,3-butanedione monoxime), and Penicillin-Streptomycin, then plated on laminin-coated (15 µg mL^-1^) dishes for 1 hour at 37 °C in 5% CO_2_. Media was then changed to culture media with or without 10 μM Angiotensin II (#ab120183, Abcam, Cambridge, UK) for 24 h. Culture media comprised M199 media supplemented with 0.1% BSA, 1% Insulin-Transferrin-Selenium (#I3146, Merck, Darmstadt, Germany), 10 mM BDM, 1% CD lipid (#11905, Thermo Fisher Scientific), and Penicillin-Streptomycin.

### Mitochondrial respiration assay

Cardiomyocytes were seeded at 4 × 10³ cells/well on Seahorse 96-well XF Cell Culture Microplates (Agilent Technologies, CA, USA) pre-coated with laminin (15 µg mL^-1^). Prior to the assay, culture media was replaced with Seahorse XF Media supplemented with 10 mM glucose, 2 mM glutamate and 1 mM sodium pyruvate, and mitochondrial function was assessed using the Seahorse XF Cell Mito Stress Test Kit (Agilent Technologies). Compounds were sequentially injected at the following final concentrations: oligomycin (2.5 μM), FCCP (1 μM), and a mixture of antimycin A and rotenone (5 μM each). Mitochondrial respiration parameters were determined as previously described^42^ and normalised to protein content.

### Statistics and reproducibility

All data represent biological replicates from at least three independent animals. For in vivo studies, each individual animal was considered one biological replicate. For in vitro assays, technical repeats were averaged to yield one value per animal (one biological repeat). Statistical analysis was performed using GraphPad Prism 10.1.2, with data expressed as mean ± SEM. Normality was assessed using Shapiro-Wilk test (*n* < 8) or D’Agostino and Pearson test (*n* ≥ 8). For normally distributed data, two-tailed unpaired Welch’s t-tests were used for two-group comparisons, while one-way ANOVA with Tukey post hoc test was applied for multiple groups. Non-normally distributed data were analysed using Mann-Whitney U tests for two groups. A *P*-value of <0.05 was considered statistically significant.

### Reporting summary

Further information on research design is available in the Nature Portfolio Reporting Summary linked to this article.

## Supplementary information

Supplementary Information

Description of Additional Supplementary files

Supplementary Data 1

Reporting Summary

## Data Availability

The RNA-seq data generated in this study has been deposited in the NCBI Sequence Read Archive (SRA) under BioProject accession PRJNA1290862. All source data can be found in Supplementary Data 1.
