# Supplementary material for: Cardiometabolic adaptations in the cave nectar bat Eonycteris spelaea
Source: Commun Biol. 2026 Mar 10;9:569. doi: 10.1038/s42003-026-09792-8 (PMC13106829; doi:10.1038/s42003-026-09792-8)
Supplement: Supplementary file 1 — Supplementary Information [file 42003_2026_9792_MOESM1_ESM.pdf]

Cardiometabolic adaptations in the cave nectar bat *Eonycteris spelaea*

Fan Yu[1-3], Akshamal M Gamage[4], Myu Mai Ja Kp[1], Randy Foo[4], Ying-Hsi Lin[1,2], Lijin Wang[5], Chee Jian Pua[1], Wharton Chan[4], Gustavo E Crespo-Avilan[1,2], Edgar M Pena[6], Lewis Z Hong[7], Aditya Iyer[8], Sujoy Ghosh[5,9], Elisa A Liehn[1], Jean-Paul Kovalik[2], Lin-Fa Wang[4], Chrishan J Ramachandra[1,2†], Derek J Hausenloy[1-3,10†*]

[†] These authors jointly supervised this work

[1]National Heart Research Institute Singapore, National Heart Centre Singapore, Singapore, Singapore
[2]Cardiovascular & Metabolic Disorders Programme, Duke-NUS Medical School, Singapore, Singapore
[3]Yong Loo Lin School of Medicine, National University of Singapore, Singapore, Singapore
[4]Programme in Emerging Infectious Diseases, Duke-NUS Medical School, Singapore, Singapore
[5]Centre for Computational Biology, Duke-NUS Medical School, Singapore, Singapore
[6]SingHealth Experimental Medicine Centre and National Large Animal Research Facility, Singapore, Singapore
[7]Paratus Sciences Singapore Pte Ltd, Singapore, Singapore
[8]Excelra, NSL Arena, Uppal, Hyderabad, India
[9]Pennington Biomedical Research Center, Baton Rouge, Louisiana, USA
[10]The Hatter Cardiovascular Institute, University College London, London, UK

Running title: Cardiometabolic adaptations in bats

*Corresponding author:

Professor Derek J Hausenloy
Cardiovascular & Metabolic Disorders Programme
Duke-NUS Medical School
8 College Road,
Singapore 169857
Email: derek.hausenloy@duke-nus.edu.sg

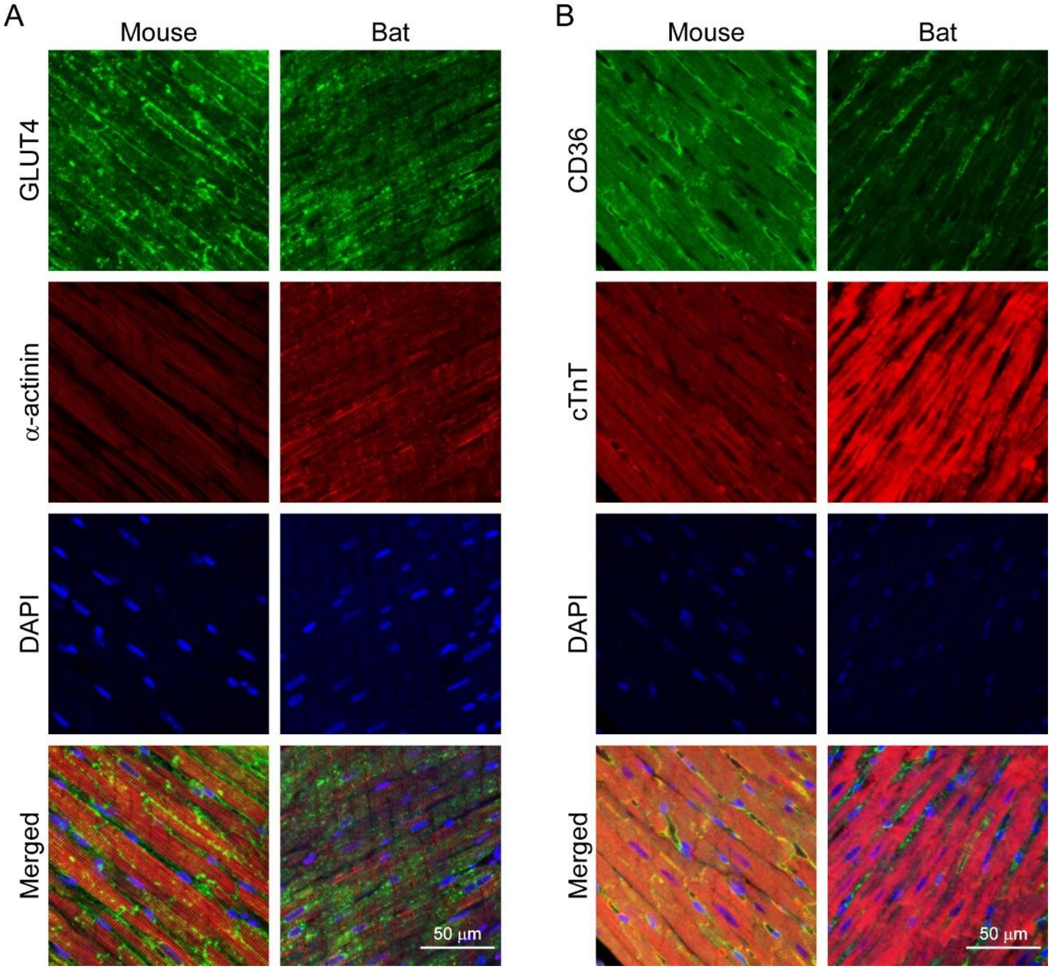

Supplementary Figure 1: (A) Single-channel and merged immunofluorescence images of bat and mouse cardiac tissue stained with GLUT4, α-actinin, and DAPI. (B) Single-channel and merged immunofluorescence images of bat and mouse cardiac tissue stained with CD36, cardiac troponin T (cTnT), and DAPI.

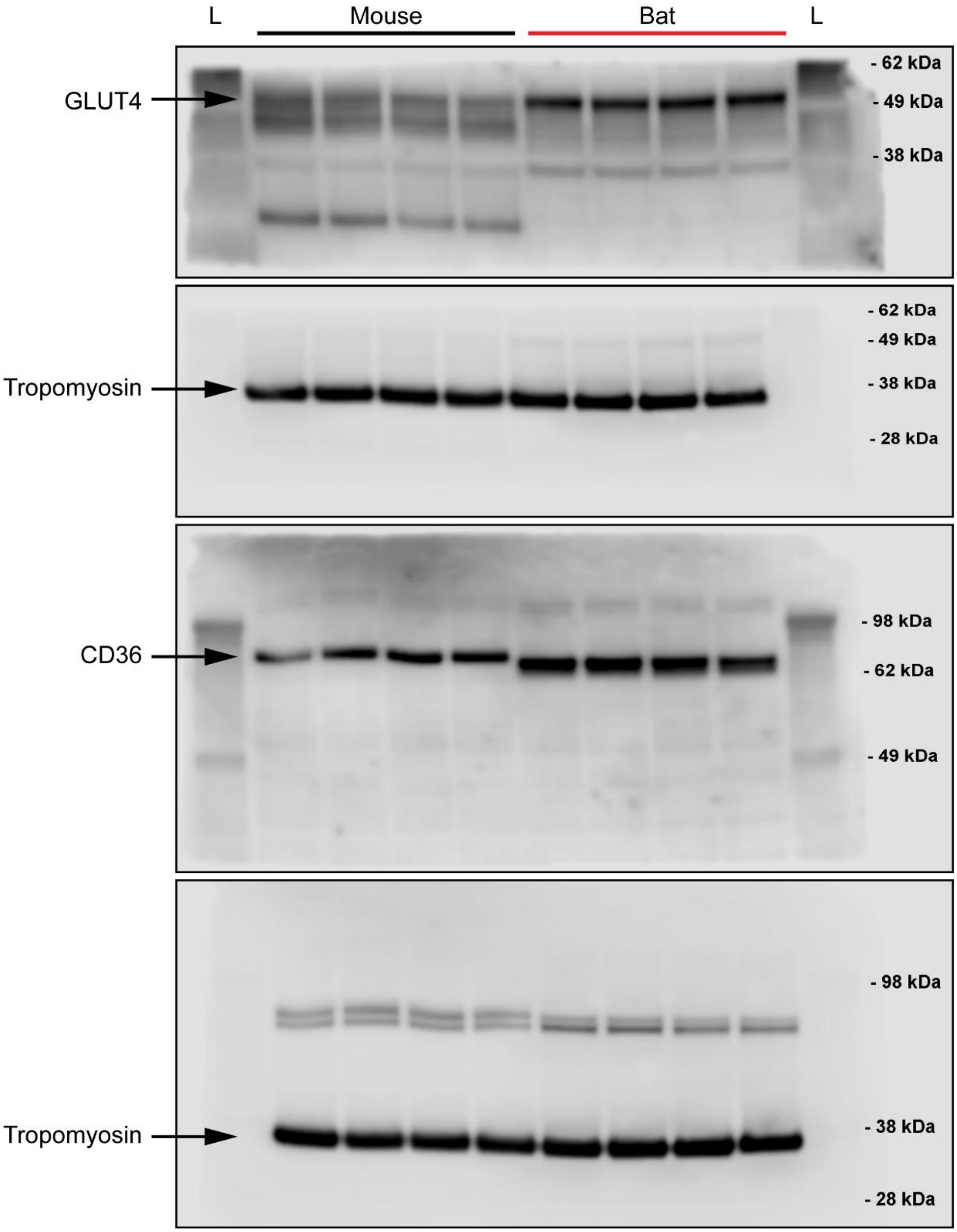

Supplementary Figure 2: Uncropped immunoblots for detecting GLUT4 and CD36 protein levels in bat and mouse cardiac tissue. Tropomyosin was used as a loading control. Abbreviations: L-molecular weight ladder.

CD36 protein sequences

Identities (82%) Positives (91%) Gaps (0%)

Mus musculus
>AAH10262.1 Cd36 protein [Mus musculus]
MGCDRNCGLIAGAVIGAVLAVFGGILMPVGDMLIEKTIKREVVLEEGTTAFKNWVKTGTTVYRQFWIFDVQNPDDVAKN
SSKIKVKQRGPYTYRVRYLAKENITQDPEDHTVSFVQPNGAIFEPSLSVGTEDDNFTVLNLAVAAAPHIYQNSFVQVVL
NSLIKKSKSSMFQTRSLKELLWGYKDPFLSLVPYPISTTVGVFYPYNDTVDGVYKVFNGKDNISKVAIIESYKGKRNLSY
WPSYCDMINGTDAASFPPFVEKSRTLRFFSSDICRSIYAVFGSEIDLKGIPVYRFVLPANAFASPLQNPDNHCFCTEKVI
SNNCTSYGVLDIGKCKEGKPVYISLPHFLHASPDVSEPIEGLHPNEDEHRTYLDVEPITGFTLQFAKRLQVNILVKPARKI
EALKNLKRPYIVPILWLNETGTIGDEKAEMFKTQVTGKIKLLGMVEMALLGIGVVMFVAFMISYCACKSKNGK

E. spelaea
MGCDRNCGLIAGAVIGAVLAVFGGILMPVGDMIIAKTITKEVVLEEGTTAFKNWVKTGTAVYRQFWIFDVQNPEEVVINS
SAIKVKQRGPYTYRVRFLAKENITHNPENNTVSFVQPRGAIFEPSLSSGTEDDTFTVLNLAVAAAPHLYPNAFVQVLLN
SLIKKSKSSMFQTRTVKELLWGYTDPFLSLVPYPVPTTVGAFFPYNNTADGVYTVFNGKDDVSKVAIIDIYKGNKNLGY
WSSYCDMVNGTDAASFPPFVEKTRILQFFSSDICRSIYAVFGAEHDLKGIPVYRFILPPEAFASPLQNPDNHCFCVDRE
VSNNCTFYGVLDISKCKGGKPVFISLPHFLHASPEITRNIEGLNPNEEEHSTYLDVEPITGFTLQFAKRLQINILVKPAKKI
EALKHLNRNYIVPVLWLNETGTIGDEKAEMFKNKVTGKVNLLGLIEIVLLSVGVVMFVAFMISYCACRSKKSK

GLUT4 protein sequences

Identities (93%) Positives (97%) Gaps (0%)

Mus musculus
>NP_033230.2 solute carrier family 2, facilitated glucose transporter member 4 isoform 1 [Mus musculus]
MPSGFQQIGSDDGEPPRQRVTGTLVLAVFSAVLGSLQFGYNIGVINAPQKVIEQSYNATWLGRQGPGGPDSIPQGTLT
TLWALSVAIFSVGGMISSFLIGIISQWLGRKRAMLANNVLAVLGGALMGLANAAASYEILILGRFLIGAYSGLTSGLVPMY
VGEIAPTHLRGALGTLNQLAIVIGILVAQVLGLESMLGTATLWPLLLALTVLPALLQLILLPFCPESPRYLYIIRNLEGPARKS
LKRLTGWADVSDALAELKDEKRKLERERPMSLLQLLGSRTHRQPLIIAVVLQLSQQLSGINAVFYYSTSIFESAGVGQPA
YATIGAGVVNTVFTLVSVLLVERAGRRTLHLLGLAGMCGCAILMTVALLLLERVPAMSYVSIVAIFGFVAFFEIGPGPIPWF
IVAELFSQGPRPAAMAVAGFSNWTCNFIVGMGFQYVADAMGPYVFLLFAVLLLGFFIFTFLKVPETRGRTFDQISAAFR
RTPSLLEQEVKPSTELEYLGPDEND

E. spelaea
MPSGFQQIGSEEGEPPQQQVTGTLVLSVFSAVLGSLQFGYNIGVINAPQKVIEQSYNETWLGRQGPEGPSSIPPGTLT
TLWALSVAIFSVGGMISSFLIGVISQWLGRKRAMLVNNTLAVLGGTLMGLANAASSYEMLILGRFLIGAYSGLTSGLVPM
YVGEISPTHLRGALGTLNQLAIVIGILIAQVLGLESMLGTAILWPLLLGITVLPALLQLVLLPFCPESPRYLYIIRNLEGPAKK
SLKRLTGWADVSGALAELKEEKRKLERERPLSLLQLLGSRTHRQPLVIAIVLQLSQQLSGINAVFYYSTSIFETAGVGQP
AYATIGAGVVNTVFTLVSVFLVERAGRRTLHLLGLAGMCGCAILMTVALLLLERFPAMSYVSIIAIFGFVAFFEIGPGPIPW
FIVAELFSQGPRPAAMALAGFSNWTCNFLIGMCFQYIADAMGPYVFLLFAVLLLSFFIFTFLKVPETRGRTFDQISAAFH
RTPSLLEQEVKPSTELEYLGPDEND

Supplementary Figure 3: Alignment of mouse and bat protein sequences for CD36 and GLUT4, illustrating the degree of sequence similarity between species. *Identities* denote amino acid residues that are identical in both sequences, while *positives* represent residues with similar physicochemical properties that likely preserve functional similarity. The absence of *gaps* indicates that no insertions or deletions occurred between the aligned sequences, suggesting conserved protein length and structure.

Supplementary Table 1: Primer sequences used in this study

Species	Gene	Forward primer sequence	Reverse primer sequence
Mouse (*Mus musculus*)	*GAPDH*	AGGTCGGTGTGAACGGATTT	ATGAAGGGGTCGTTGATGGC
	SLC2A4	GCGCCTACTCAGGGCTAAC	TGGCCAGTTGGTTGAGTGTT
	CD36	ATTAATGGCACAGACGCAGC	TTCAGATCCGAACACAGCGT
Bat (*Eonycteris spelaea*)	*GAPDH*	CATCAAATGGGGCGATGCTG	CATCCGCAGAAGGAGCAGAA
	SLC2A4	AGGGTAGAAAAAGCCCCTGC	TCACCCTGCACAACCTAACC
	CD36	ACCCACAATCCTGAGAACAACA	CAGCAGCAACAGCCAGATTG
