# Supplementary material for: Cardiometabolic adaptations in the cave nectar bat Eonycteris spelaea
Source: Commun Biol. 2026 Mar 10;9:569. doi: 10.1038/s42003-026-09792-8 (PMC13106829; doi:10.1038/s42003-026-09792-8)
Supplement: Supplementary file 2 — Description of Additional Supplementary files [file 42003_2026_9792_MOESM2_ESM.pdf]

Description of Additional Supplementary files

File name: Supplementary Data 1

Description: Source data for figures used in the study.
